# Hepatic splenosis mimicking HCC in a patient with hepatitis C liver cirrhosis and mildly raised alpha feto protein; the important role of explorative laparoscopy

**DOI:** 10.1186/1477-7819-7-1

**Published:** 2009-01-05

**Authors:** M Abu Hilal, A Harb, B Zeidan, B Steadman, JN Primrose, NW Pearce

**Affiliations:** 1 Hepatobiliary-Pancreatic and Laparoscopic Surgical Unit, Southampton University Hospital, Southampton, SO16 6YD, UK

## Abstract

**Background:**

Splenosis is a heterotropic implantation of splenic fragments onto exposed vascularised peritoneal and intrathoracic surfaces, following splenic injury or elective splenectomy.

**Case presentation:**

A 60 year old cirrhotic patient was referred to us with a hepatic mass, suspected to be HCC in a cirrhotic liver. A computerized tomography scan (CT) demonstrated a cirrhotic liver with a 2 × 2.7 cm focal hypervascular nodule, lying peripherally at the junction of segment 7 and 8. Diagnostic laparoscopy demonstrated a 3 cm exofitic dark brown splenunculus attached to the diaphragm and indenting the surface of segment 7 of the liver. The lesion was easily resected laparoscopically and shaved from the live surface with no need for a liver resection. The histopathological assessment confirmed the diagnosis of splenunculus, with no evidence of neoplasia.

**Conclusion:**

Hepatic splenosis is not a rare event and should be suspected in patients with a history of splenic trauma or splenectomy. Correct diagnosis is essential and will determine subsequent management plans. In doubtful cases laparoscopic investigation can offere essential information and should be part of the standard protocol for investigating suspected splenosis.

## Background

Splenosis is a heterotropic implantation of splenic fragments onto exposed vascularised peritoneal and intrathoracic surfaces, following splenic injury or elective splenectomy.

There are a few reported cases of hepatic splenosis in the English literature, which has usually a challenging and difficult differential diagnosis with hepatic adenoma, haemangioma, focal nodulal hyperplasia, lymphoma and hepatocellular carcinoma [1-4]. Excluding a diagnosis of Hepato cellular carcinoma (HCC) has proven difficult despite different suggested radiological investigation methods [5,6], particularly in patients with chronic liver disease [7-11], when HCC is an expected development and a more likely underlying pathology than is intrahepatic splenosis.

Laparoscopic exploration provides a port of minimally invasive entry for the visualisation of suspect masses, and allows access for potential subsequent biopsy or resection. To our knowledge, this is the first case were explorative laparoscopy has been an essential tool to confirm the diagnosis of splenosis and rule out the doubt of malignancy. This had a significant positive impact on this patient's management, avoiding unneccassary laparotomy or'/and surgical resection in a high-risk patient.

Hepatic splenosis is not a rare event and should be suspected in patients with a history of splenic trauma or splenectomy. Correct diagnosis is essential and will determine subsequent management plans. In doubtful cases; laparoscopic investigation can offer essential information and should be part of the standard protocol for investigating suspected splenosis.

## Case presentation

A 60 year old cirrhotic patient was referred to us with a hepatic mass, suspected to be HCC in a cirrhotic liver. The patient was diagnosed with liver cirrhosis in December 2003, secondary to Hepatitis C infection after receiving blood transfusions at splenectomy for a ruptured spleen 46 years ago.

Serial blood tests showed sudden derangement in his liver function, and a mild rise in alpha-feto protein levels. Clinically, he complained of non-specific flu-like symptoms and also reported recent weight loss and reduced appetite.

On examination he appeared jaundiced, there were signs of clubbing and on examination of his abdomen, there was a mild degree of ascites and the liver edge was palpable.

**Figure 1:**
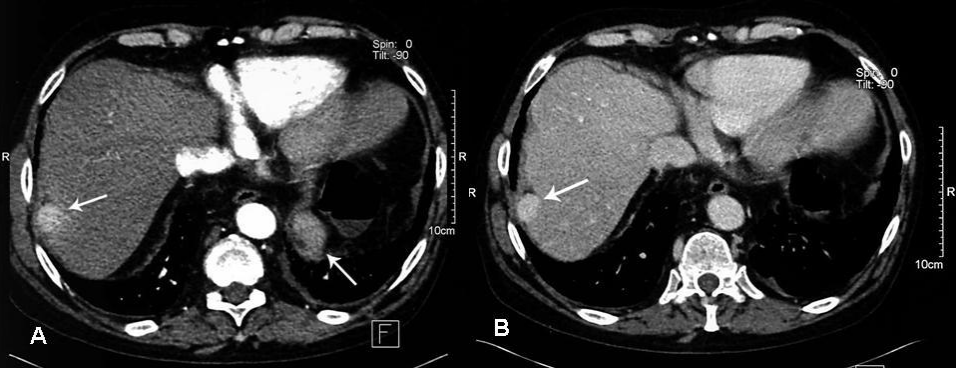
**CT scan**. (A) Axial IV contrast enhanced CT. Arterial phase image showing a 2 × 2.7 cm hypervascular, subcapsular nodule in segment VII of the liver. (B) Portal venous phase image.

He was a persistent alcoholic and was prone to frequent binging. A computerized tomography scan (CT) demonstrated a cirrhotic liver with a 2 × 2.7 cm focal hypervascular nodule, lying peripherally at the junction of segment 7 and 8 (figure 1). There was an increased enhancement in the venous phase scans, and the picture was very suspicious for a focal hepatoma.

**Figure 2:**
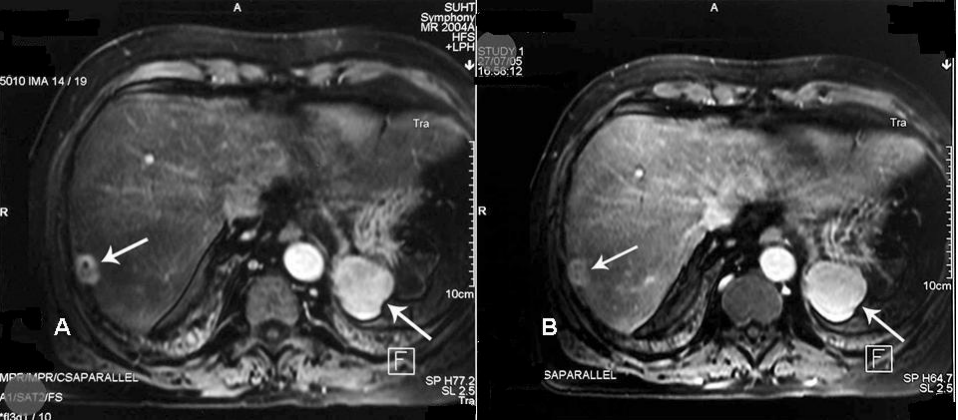
**Arterial (A) and portal venous phase (B) of IV Gadlinium enhanced axial MRI images demonstrating a solitary 2 cm hypervascular nodule in segment VII (Arrow)**. A 4.5 cm nodule with similar enhancement characteristics is also noted in the left upper quadrant (Arrow).

**Figure 3:**
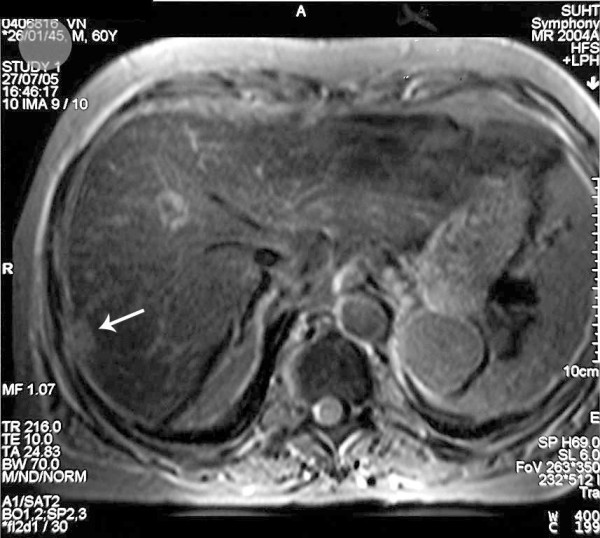
**Post Resovet Axial MRI images**. The segment VII lesion demonstrates higher signal than the background, reflecting a relative lack of functioning hepatocytes.

A double contrast MR study, using Gadolinium and resovist contrasts, confirmed the presence of a solitary 2 × 2.5 cm mass with features suggestive of hepatoma, lying within segment 7 in a subcapsular position (figures 2 &3). Although a similar characteristics 4.5 cm mass was also noted in the left upper quadrant; malignancy couldn't be excluded. The multi-disciplinary meeting advised an explorative laparoscopy for further investigation of this lesion, and better assessment of the extent of the disease.

Diagnostic laparoscopy demonstrated a 3 cm exofitic dark brown splenunculus attached to the diaphragm and indenting the surface of segment 7 of the liver. Multiple other typical looking splenunculi were found. Intraoperative ultrasound was performed and excluded any other lesions within the liver or surrounding tissues.

The lesion was easily resected laparoscopically and shaved from the live surface with no need for a liver resection. The histopathological assessment confirmed the diagnosis of splenunculus, with no evidence of neoplasia.

The patient finished a 24 weeks course of pegylated interferon α and ribavirin as part of his hepatitis C treatment regime post operatively. At two years his follow up showed no radiologic evidence of HCC, and his LFTs and AFP were within normal limits.

## Discussion

Splenosis is the heterotropic implantation of splenic fragments onto exposed vascularised peritoneal and intrathoracic surfaces, following splenic injury or elective splenectomy. This can occur anywhere within the abdominal cavity and the resultant splenunculus will receive its blood by parasitizing the surrounding tissue.

There are few previous reports of hepatic splenosis mimicking hepatocellular carcinoma [10,12,13]. In most cases, correct diagnosis was only possible on histological examination after a laparotomy and open liver resection [10,14]. A missed diagnosis of hepatic splenosis can have a significant negative impact on patient's management [15]. Interestingly, in all cases a history of post-traumatic splenectomy was reported and all patients were known to have an underlying chronic liver disease [10,16,17]. There are no typical radiological features of intrahepatic splenosis and it is usually difficult to distinguish this condition from other liver tumors. In the presence of chronic liver disease, although mild but raised tumoral markers and strong suspicion of HCC on clinical ground, establishing the correct diagnosis can prove to be difficult.

Distinguishing the nature of a hepatic mass is important because it significantly alters patient management. In this case, if a diagnosis of HCC was confirmed, this patient would be suitable for resection (Child-Pugh class B) or for a liver transplant, satisfying the Milan criteria. Liver cirrhosis, having recent LFT derangement and with the above radiological picture made HCC strongly suspected. However, the pervious history of traumatic splenic rupture and the presence of multiple splenunculi within the abdominal cavity suggested that the best way to proceed would be for a laparoscopic exploration and a secure diffinition of the lesions nature before planning for future management.

Laparoscopy was sufficient in confirming diagnosis of splenosis, as well as excluding coexistent malignancy. This had a significant impact of clinical plans and patients management. It is already recognized that laparoscopy provides a port of minimally invasive entry for the visualisation of suspect masses, and allows access for potential subsequent biopsy or resection.

The abnormal liver function behavior in this case can be explained by an active hepatitis C process, which have improved following further anti viral treatment. Yet, a low threshold for HCC is a must with any similar scenario of suspicious liver function tests and radiological findings.

Laparoscopic resection of symptomatic or suspicious splenosis is a minimally invasive and feasible procedure. This was reported to be a successful diagnostic and interventional tool even in laparoscopically challenging scenarios involving the pancreas [18,19].

To the best of our knowledge, this is the first case where laparoscopy has been the main tool in difining the correct diagnosis in a case of splenosis, suspected to be an HCC on radiological investingations and strong clinical bases.

We therefore propose that laparoscopic investigation should be part of a new approach for investigating suspect intrahepatic masses.

## Conclusion

Hepatic splenosis is not a rare event and should be considered with the differential diagnosis in case of suspected lesions especially in patients who had previous splenectomy. Correct diagnosis is essential and can significantly influence patient management. We propose that laparoscopic investigation should be part of a new protocol for confirming the diagnosis of suspected intrahepatic masses.

## Consent

Written informed consent was obtained from the patient for publication of this case report and accompanying images. A copy of the written consent is available for review by the Editor-in-Chief of this journal.

## Competing interests

The authors declare that they have no competing interests.

## Authors' contributions

MAH wrote the paper. AH was responsible for literature review, medline search and wrote the first draft. BZ wrote the case history and collected all clinical information. JNP reviewed the article and made suggestions. NWP was the surgeon, reviewed the paper and made suggestions. BS was the radiologist who selected the images and commented on the manuscript.
